# Artificial Intelligence–Generated Draft Replies to Patient Inbox Messages

**DOI:** 10.1001/jamanetworkopen.2024.3201

**Published:** 2024-03-20

**Authors:** Patricia Garcia, Stephen P. Ma, Shreya Shah, Margaret Smith, Yejin Jeong, Anna Devon-Sand, Ming Tai-Seale, Kevin Takazawa, Danyelle Clutter, Kyle Vogt, Carlene Lugtu, Matthew Rojo, Steven Lin, Tait Shanafelt, Michael A. Pfeffer, Christopher Sharp

**Affiliations:** 1 Department of Medicine, Stanford University School of Medicine, Stanford, California; 2 Stanford Healthcare AI Applied Research Team, Division of Primary Care and Population Health, Stanford University School of Medicine, Stanford, California; 3 Department of Family Medicine, University of California San Diego School of Medicine, La Jolla; 4 Technology and Digital Solutions, Stanford Medicine, Stanford, California; 5 Nursing Informatics & Innovation, Stanford Healthcare, Stanford, California; 6 WellMD Center, Stanford University School of Medicine, Stanford, California

## Abstract

**Importance:**

The emergence and promise of generative artificial intelligence (AI) represent a turning point for health care. Rigorous evaluation of generative AI deployment in clinical practice is needed to inform strategic decision-making.

**Objective:**

To evaluate the implementation of a large language model used to draft responses to patient messages in the electronic inbox.

**Design, Setting, and Participants:**

A 5-week, prospective, single-group quality improvement study was conducted from July 10 through August 13, 2023, at a single academic medical center (Stanford Health Care). All attending physicians, advanced practice practitioners, clinic nurses, and clinical pharmacists from the Divisions of Primary Care and Gastroenterology and Hepatology were enrolled in the pilot.

**Intervention:**

Draft replies to patient portal messages generated by a Health Insurance Portability and Accountability Act–compliant electronic health record–integrated large language model.

**Main Outcomes and Measures:**

The primary outcome was AI-generated draft reply utilization as a percentage of total patient message replies. Secondary outcomes included changes in time measures and clinician experience as assessed by survey.

**Results:**

A total of 197 clinicians were enrolled in the pilot; 35 clinicians who were prepilot beta users, out of office, or not tied to a specific ambulatory clinic were excluded, leaving 162 clinicians included in the analysis. The survey analysis cohort consisted of 73 participants (45.1%) who completed both the presurvey and postsurvey. In gastroenterology and hepatology, there were 58 physicians and APPs and 10 nurses. In primary care, there were 83 physicians and APPs, 4 nurses, and 8 clinical pharmacists. The mean AI-generated draft response utilization rate across clinicians was 20%. There was no change in reply action time, write time, or read time between the prepilot and pilot periods. There were statistically significant reductions in the 4-item physician task load score derivative (mean [SD], 61.31 [17.23] presurvey vs 47.26 [17.11] postsurvey; paired difference, −13.87; 95% CI, −17.38 to −9.50; *P* < .001) and work exhaustion scores (mean [SD], 1.95 [0.79] presurvey vs 1.62 [0.68] postsurvey; paired difference, −0.33; 95% CI, −0.50 to −0.17; *P* < .001).

**Conclusions and Relevance:**

In this quality improvement study of an early implementation of generative AI, there was notable adoption, usability, and improvement in assessments of burden and burnout. There was no improvement in time. Further code-to-bedside testing is needed to guide future development and organizational strategy.

## Key Points

**Question:**

What is the adoption of and clinician experience with clinical practice deployment of a large language model used to draft responses to patient inbox messages?

**Findings:**

In this 5-week, single-group, quality improvement study of 162 clinicians, the mean draft utilization rate was 20%, there were statistically significant reductions in burden and burnout score derivatives, and there was no change in time.

**Meaning:**

These findings suggest that the use of large language models in clinical workflows was spontaneously adopted, usable, and associated with improvement in clinician well-being.

## Teaser

This quality improvement study evaluates a large language model used to draft responses to patient messages in terms of utilization, usability, utility, impact on clinician well-being, and time spent in the electronic inbox.

## Introduction

The emergence and ubiquity of generative artificial intelligence (AI) represents a turning point for health care. As organizations consider how to approach this new technology, several questions must be answered: How can the highest value and lowest risk use cases be identified? How should clinical practice deployments be structured to determine value? When is the right time to invest in widespread adoption of these technologies? As organizations grapple with how to integrate and implement AI technologies in a fair, useful, and reliable way,^1^ rigorous evaluation of clinical practice generative AI deployment is needed to inform strategic decision-making.

One potentially high-value use case for large language models is to help address patient portal messaging, which has seen a 157% increase during the COVID-19 pandemic compared with prepandemic levels.^2,3,4^ This rapidly growing modality for care has strained health system capacity and become a leading factor in clinician burnout.^2,3^ Although several strategies have been proposed for inbox management, including automated message categorization and triage, team optimization, and billing for patient messages,^5,6,7^ more effective solutions are required.

Large language models, like generative pretrained transformer 4 (GPT-4; OpenAI),^8^ have shown the ability to draft empathetic responses to online patient questions^9^ and to assist with medical knowledge tasks.^10^ A novel Health Insurance Portability and Accountability Act–compliant, electronic health record (EHR)–integrated version of this tool was created to generate draft replies to patient portal messages for clinicians but has not been assessed in clinical practice.^11^ Therefore, this study evaluated the implementation of this novel technology using an evaluation guided by the Reach, Efficacy, Adoption, Implementation, Maintenance/Practical, Robust Implementation, and Sustainability Model (RE-AIM/PRISM).^12,13,14^ The developmental assessment of readiness for adoption and clinician experience included a primary outcome of utilization, with secondary outcomes evaluating time, usability, utility, and impact on clinician well-being.

## Methods

 The Stanford University institutional review board office determined that this study met the criteria for quality improvement and was exempt from institutional review board–mandated consent. All clinicians who participated were directly contacted via email and were allowed to opt out of the pilot. Clinicians who completed the presurvey or postsurvey were compensated with a $20 gift card upon completion of the postsurvey. Standards for Quality Improvement Reporting Excellence (SQUIRE) reporting guidelines for quality improvement studies were followed.

### Large Language Model Integration

Stanford Medicine routed select patient messages upon arrival to the inbox messaging pool to EHR developer Epic (Epic Systems) for categorization via GPT-3.5 Turbo (selected by EPIC to minimize cost and compute) and GPT-4 for draft reply generation (eFigure 1 in Supplement 1). Messages written in a non-English language, with an attachment, or sent by a proxy were excluded. Messages were categorized into 1 of 4 categories (general, results, medications, and paperwork), which triggered a corresponding prompt that included the patient message, selected structured data elements (eg, name, age, department, allergies, and so forth) and the last clinic note. Patient messages and draft replies were displayed within the EHR with options to start with draft or start blank reply (eFigure 2 in Supplement 1). Technical reliability was tested with 14 ambulatory clinician beta users before study initiation. Education was provided via email and brief presentations at clinician group meetings.

### Study Design, Setting, Participants, and Data Collection

This prospective quality improvement study was conducted from July to August 2023 at a single academic medical center (Stanford Health Care). All attending physicians, advanced practice practitioners (APPs), clinical nurses, and clinical pharmacists from the Division of Primary Care and the Division of Gastroenterology and Hepatology were invited to participate. The pilot period lasted for 35 days extending from July 10 through August 13, 2023. A corresponding 35-day prepilot period extending from May 29 through July 2, 2023, was used for comparison.

### Study Measures

The RE-AIM/PRISM framework was used to evaluate the implementation from the perspective of clinicians (eTable 1 in Supplement 1). For utilization, view was defined as any time a patient message was viewed by a pilot clinician; other action as any action taken by a pilot clinician directly linked to the message other than a reply (eTable 2 in Supplement 1); reply action as instances where pilot clinicians clicked start with draft, start blank reply, or reply to patient; and draft used as instances where pilot clinicians clicked start with draft. Patient messages that were not viewed by a pilot clinician and actions not linked to a message were not included in the analyses (eFigure 1 in Supplement 1).

For the time analysis, other action time was defined by the time between when the user last viewed the message and when the action on the message was initiated. Read time was measured as the time between when the user last viewed the message and when the user clicked start with draft, start blank reply, or reply to patient. Write time was measured as the time between when the user clicked start with draft, start blank reply, or reply to patient and when the reply was sent. Reply action time was defined by the time between when the user last viewed the message and when the message was sent. All times were measured in seconds using audit log data (eFigure 3 in Supplement 1). Outliers above 3000 seconds (0.1% of other action times, 1.23% of read times, and 0.26% of write times) were excluded.

Presurveys and postsurveys were distributed to all clinicians via email. Specialty and role were captured for all pilot users with additional demographic data captured for survey respondents. EHR burden was evaluated using an adapted NASA Task Load Index score with a 4-item physician task load score derivative.^15,16,17^ Physician task load scores range from 0 to 100, with lower scores indicating less cognitive task load. Burnout was evaluated using the 4-item questionnaire evaluating work exhaustion from the Stanford Professional Fulfillment Index (PFI-WE). Work exhaustion scores range from 0 to 4, with lower scores indicating lower levels of work exhaustion.^18,19^ Satisfaction was assessed via net promoter score, which is calculated by categorizing likelihood to recommend responses into promoters (score 9-10), passives (score 7-8), and detractors (score 0-6) and then subtracting the percentage of detractors from the percentage of promoters. The net promoter score ranges from –100 to 100, with higher scores indicating higher levels of satisfaction.^20^ Usability was assessed with questions regarding perceived utility, quality, and time. Likert scale scores for utility, quality, and time range from 1 to 5, with lower scores indicating a higher level of agreement on a 5-point Likert scale. The text of all survey questions is available in eFigure 4 in Supplement 1.

Free-text survey comments were analyzed to assess clinician perspectives. Comments were parsed into phrases to allow for granular analysis. A comprehensive code book, rooted in RE-AIM/PRISM, was developed to guide the systematic analysis. Both deductive and inductive codes were included. Two qualitative researchers (M.S. and A.D.S.) independently coded phrases using the deductive code book followed by consensus reconciliation. During consensus coding, inductive codes were introduced to capture emerging themes. Phrases were allowed to have multiple codes, and each code was assigned a positive, negative, or neutral connotation. Counts of codes in each category, as well as the distribution of sentiments (positive, negative, and neutral), were aggregated and summarized.

### Statistical Analysis

Clinician demographics were described with counts and proportions. A χ^2^ test was used to compare the distribution of specialties and roles in the overall cohort compared with the survey cohort, with statistical significance considered at *P* < .05.

Counts for reply actions, reply actions with draft available, and drafts used were aggregated at the level of individual clinicians for the pilot period. Draft utilization for each clinician was calculated as the count of drafts used by that clinician divided by the number of reply actions with drafts available. Summary statistics were calculated as means and SDs. To assess for significant between-group differences in draft utilization, the Kruskal-Wallis test was used, with *P* < .05 considered statistically significant. Counts and proportions for draft availability were calculated for views, actions, and replies in the pilot period. For replies, counts and proportions were also calculated for message exclusions and error types.

The mean other action time, reply action time, read time, and write time was calculated for each clinician in both the prepilot and pilot periods. For those with at least 1 action in both periods, the change in mean other action time was calculated. For those with at least 1 reply in both periods, the change in mean reply action time, read time, and write time was also calculated. All measures were summarized as means and SDs. One-sample *t* tests were used to compare the observed differences against a null hypothesis of no change between the prepilot and pilot periods. *P* < .05 was considered statistically significant. To account for repeated measures and heterogeneity across clinicians, analysis was also performed using linear mixed effects models. The intervention period (ie, prepilot vs pilot) was considered as a fixed effect with clinicians incorporated as random effects with both a random intercept and random slope. A logarithmic transform was applied to time measures before fitting the model. *P* < .05 was considered significant.

The analysis of survey responses used the Wilcoxon signed-rank test, and statistical significance was considered for 2-sided *P* < .05. Analysis was performed using the statsmodels package in Python programing language version 3.11.4 (Python Software Foundation), with the exception of the survey data, which were analyzed using Minitab Statistical Software version 21.4.2.0 (Minitab, LLC). Prespecified subgroups included specialties and roles.

## Results

Of the 197 clinicians enrolled in the pilot, 162 were included in the study analysis. Clinicians who were in the prepilot (14 clinicians), out of office (16 clinicians), or not working in a specific ambulatory clinic (4 triage nurses and 1 procedural nurse) were excluded (eFigure 5 in Supplement 1). The survey group consisted of 73 participants (45.1%) who completed both the presurvey and postsurvey.

In gastroenterology and hepatology, there were 58 physicians and APPs and 10 nurses. In primary care, there were 83 physicians and APPs, 4 nurses, and 8 clinical pharmacists (Table 1). There was no statistically significant difference in the distribution of specialties and roles between the overall cohort and the survey cohort (χ^2^_4_ = 7.96; *P* = .09). Additional demographics for the survey group, including age, years in practice since training, gender, and number of half-days of clinic per week, are also shown.

**Table 1.** Cohort Demographics

Characteristic	Participants, No. (%)	
	Overall cohort (N = 162)	Survey cohort (n = 73)
Specialty and role		
Primary care	95 (58)	41 (56)
Physician and APP	83 (51)	30 (41)
Nurse	4 (2)	4 (5)
Clinical pharmacist	8 (5)	7 (10)
Gastroenterology and hepatology	68 (42)	32 (44)
Physician and APP	58 (36)	22 (30)
Nurse	10 (6)	10 (14)
Age, y^a^		
25-34	NA	20 (27)
35-44	NA	33 (45)
45-54	NA	13 (18)
55-64	NA	6 (8)
≥65	NA	1 (1)
Years after training^b^		
0-4	NA	25 (34)
5-9	NA	15 (21)
10-14	NA	16 (22)
≥15	NA	17 (23)
Gender^c^		
Female	NA	57 (78)
Male	NA	15 (21)
Nonbinary	NA	1 (1)
Half-days in clinic^d^		
0	NA	11 (15)
1	NA	3 (4)
1.5	NA	2 (3)
2	NA	8 (11)
3	NA	9 (12)
4	NA	14 (19)
5	NA	4 (5)
6	NA	9 (12)
7	NA	7 (10)
8	NA	4 (5)
9	NA	2 (3)

Abbreviations: APP, advanced practice practitioner; NA, not applicable.

^a^ Age was self-reported. Responses were mutually exclusive. Percentages sum to 99 owing to rounding artifact.

^b^ Years after training was self-reported. Responses were mutually exclusive.

^c^ Gender was self-reported. Responses were mutually exclusive and included the option of “Prefer not to answer.”

^d^ Half-days in clinic were self-reported as free-text responses. Percentages sum to 99 owing to rounding artifact.

### Adoption and Utilization

The overall mean utilization rate per clinician was 20% (Table 2), with significant between-group differences in utilization (*k* = 10.8; *P* = .03 by Kruskal-Wallis test). For gastroenterology and hepatology, nurses had the highest utilization at 29%, whereas clinical pharmacists had the highest utilization for primary care at 44%. High SDs reflect substantial within-group variability in both reply counts and draft utilization, confirmed on visualization of the behaviors of individual clinicians (eFigures 6 and 7 in Supplement 1).

**Table 2.** Draft Utilization per Clinician Stratified by Specialty and Role

Specialty and role	Mean (SD)			
	Reply action count	Reply action count with draft available	Draft used count	Draft utilization rate
Overall	79.3 (95.5)	59.4 (72.6)	8.6 (16.9)	0.203 (0.268)
Primary care	98.5 (84.4)	74.1 (62.9)	9.3 (11.3)	0.176 (0.212)
Physician and APP	102.0 (75.5)	78.5 (61.0)	9.9 (11.9)	0.153 (0.185)
Nurse	164.8 (215.0)	97.0 (109.0)	5.0 (6.8)	0.111 (0.136)
Clinical pharmacist	29.5 (26.0)	17.4 (15.9)	5.1 (3.8)	0.444 (0.317)
Gastroenterology and hepatology	52.8 (103.9)	39.1 (80.3)	7.6 (22.6)	0.250 (0.342)
Physician and APP	19.3 (33.2)	12.9 (20.6)	1.1 (1.8)	0.240 (0.365)
Nurse	246.5 (156.3)	191.1 (123.5)	45.0 (44.2)	0.293 (0.219)

Abbreviation: APP, advanced practice practitioner.

Of the 12 844 messages for which replies were sent to patients, 9621 (75%) had a draft available (eTable 3 in Supplement 1). Of the 3223 (25%) messages for which a draft was not available, 2596 (20%) were due to exclusion criteria and 627 (5%) were due to technical limitations such as insufficient compute and token limits.

### Time Spent in the Inbox

The mean (SD) change in time spent between the prepilot and pilot periods was not significant for other action time (1.3 [27.6] seconds), reply action time (11.8 [104.6] seconds), read time (6.7 [85.6] seconds), or write time (5.1 [70.2] seconds). Clinicians who performed at least 1 action (161 clinicians) or 1 reply (138 clinicians) in the prepilot and pilot periods were included in the analysis. There were notable differences in time parameters on subgroup analyses (Table 3). On visualization of individual clinicians, there was significant within-group heterogeneity, although clinicians with higher reply counts fall closer to the diagonal, suggesting that at least part of the variation was random (eFigure 8 in Supplement 1).

**Table 3.** Change in Time Spent per Clinician on Actions

Variable	Time, mean (SD), s		
	Prepilot	Pilot	Change
Other action time			
Overall	31.8 (24.5)	33.1 (24.1)	1.3 (27.6)^a^
Primary care	30.1 (21.2)	30.6 (22.8)	0.4 (28.0)
Physician and APP	27.7 (16.4)	28.3 (17.2)	0.6 (19.0)
Nurse	71.2 (35.9)	36.6 (8.4)	−34.6 (32.7)
Clinical pharmacist	35.0 (35.6)	50.7 (53.7)	15.8 (69.3)
Gastroenterology and hepatology	34.2 (28.5)	36.6 (25.6)	2.5 (27.3)
Physician and APP	32.6 (29.0)	35.7 (25.2)	3.2 (28.4)
Nurse	43.7 (24.5)	42.0 (28.7)	−1.7 (21.0)
Reply action time			
Overall	259.7 (160.7)	267.9 (162.5)	11.8 (104.6)^b^
Primary care	228.8 (120.3)	238.8 (125.5)	11.0 (67.9)
Physician and APP	211.4 (104.5)	220.6 (110.1)	9.2 (60.8)
Nurse	491.0 (121.0)	462.8 (134.8)	−28.2 (138.0)
Clinical pharmacist	282.8 (117.4)	313.0 (149.6)	54.4 (89.4)
Gastroenterology and hepatology	315.9 (205.4)	317.6 (203.1)	13.5 (156.3)
Physician and APP	276.4 (181.2)	284.0 (181.2)	20.0 (171.3)
Nurse	478.1 (230.2)	468.9 (236.7)	−9.2 (88.8)
Read time			
Overall	113.0 (86.6)	118.3 (82.4)	6.7 (85.6)^c^
Primary care	100.1 (62.2)	108.1 (60.4)	8.0 (53.4)
Physician and APP	92.9 (55.5)	98.0 (47.3)	5.1 (50.4)
Nurse	212.1 (88.6)	206.3 (101.6)	−5.8 (72.3)
Clinical pharmacist	120.7 (65.0)	163.1 (89.8)	49.5 (67.9)
Gastroenterology and hepatology	136.5 (116.1)	135.8 (108.8)	4.1 (129.7)
Physician and APP	113.5 (103.5)	125.7 (109.4)	19.4 (139.4)
Nurse	231.1 (122.1)	181.4 (98.6)	−49.7 (67.8)
Write time			
Overall	146.7 (98.4)	149.5 (113.3)	5.1 (70.2)^d^
Primary care	128.7 (80.4)	130.6 (89.6)	3.0 (40.1)
Physician and APP	118.5 (73.8)	122.6 (87.5)	4.1 (36.8)
Nurse	278.9 (37.2)	256.5 (45.9)	−22.4 (71.5)
Clinical pharmacist	162.1 (84.3)	149.9 (82.7)	4.9 (58.2)
Gastroenterology and hepatology	179.4 (118.7)	181.8 (140.3)	9.4 (109.3)
Physician and APP	162.9 (111.2)	158.3 (110.7)	0.6 (104.0)
Nurse	247.1 (130.1)	287.5 (207.6)	40.4 (127.1)

Abbreviation: APP, advanced practice practitioner.

^a^ *P* = .56.

^b^ *P* = .19.

^c^ *P* = .36.

^d^ *P* = .40.

To account for repeated measures and the observed heterogeneity, a similar analysis was performed comparing the same time measures in the prepilot and pilot periods using linear mixed effects models (eFigure 9 in Supplement 1). No significant change in time spent was identified as a result of the intervention (eTable 4 in Supplement 1).

### Usability, Utility, and Clinician Well-Being

Table 4 delineates the pivotal components from the presurvey and postsurvey results. Statistically significant reductions in 4-item physician task load score derivative (mean [SD], 61.31 [17.23] presurvey vs 47.26 [17.11] postsurvey; paired difference, −13.87; 95% CI, −17.38 to −9.50; *P* < .001) and work exhaustion scores (PFI-WE) (mean [SD], 1.95 [0.79] presurvey vs 1.62 [0.68] postsurvey; paired difference, −0.33; 95% CI, −0.50 to −0.17; *P* < .001) were found overall. When stratified by specialty, similar reductions in physician task load and work exhaustion were seen for both primary care (task load, mean [SD], 58.72 [14.64] presurvey vs 47.72 [18.15] postsurvey; paired difference, −10.99; 95% CI, −15.00 to −5.50; *P* < .001; work exhaustion, mean [SD], 1.96 [0.65] presurvey vs 1.64 [0.64] postsurvey; paired difference, −0.33; 95% CI, −0.50 to −0.42; *P* = .009) and gastroenterology and hepatology (task load, mean [SD], 64.39 [19.50] presurvey vs 46.63 [15.27] postsurvey; paired difference, −17.76; 95% CI, −26.38 to −11.25; *P* < .001; work exhaustion, mean [SD], 1.93 [0.93] presurvey vs 1.60 [0.74] postsurvey; paired difference, −0.31; 95% CI, −0.50 to −0.13; *P* < .001). Clinicians overall expressed optimism about utility and ability to save time before the pilot, and these positive perceptions remained largely unchanged afterward (mean [SD] score, 2.15 [0.85] presurvey vs 2.17 [0.99] postsurvey for utility; 2.23 [0.82] presurvey vs 2.29 [1.20] postsurvey for time-saving). In primary care, there were modest expectations about message quality that improved at the end of the pilot (mean [SD] score, 2.73 [0.98] presurvey vs 2.23 [1.12] postsurvey; paired difference, −0.43; 95% CI, −1.00 to −0.00; *P* = .04). Net promoter scores were favorable among primary care physicians and APPs (score, 13), primary care clinical pharmacists (score, 71), and gastroenterology and hepatology nurses (score, 50), but unfavorable among primary care nurses (score, −60) and gastroenterology and hepatology physicians and APPs (score, −19).

**Table 4.** Presurvey and Postsurvey Results

Variable	Score, mean (SD)		*P* value	No. of responses
	Presurvey	Postsurvey		
Physician task load score derivative^a^				
Overall	61.31 (17.23)	47.26 (17.11)	<.001	73
Primary care	58.72 (14.64)	47.72 (18.15)	<.001	42
Physician and APP	59.11 (16.09)	50.75 (17.62)	NA	30
Nurse	54.75 (5.02)	43.90 (22.13)	NA	5
Clinical pharmacist	59.96 (12.03)	37.50 (11.92)	NA	7
Gastroenterology and hepatology	64.39 (19.50)	46.63 (15.27)	<.001	31
Physician and APP	64.74 (19.51)	48.46 (16.46)	NA	21
Nurse	63.68 (19.48)	42.80 (11.52)	NA	10
Burnout and work exhaustion score^b^				
Overall	1.95 (0.79)	1.62 (0.68)	<.001	71^c^
Primary care	1.96 (0.65)	1.64 (0.64)	.009	40
Physician and APP	1.95 (0.57)	1.63 (0.69)	NA	30
Nurse	1.58 (0.12)	1.47 (0.17)	NA	3^c^
Clinical pharmacist	2.20 (0.96)	1.75 (0.50)	NA	7
Gastroenterology and hepatology	1.93 (0.93)	1.60 (0.74)	.003	31
Physician and APP	2.02 (0.88)	1.65 (0.73)	NA	21
Nurse	1.73 (1.00)	1.48 (0.75)	NA	10
Utility^d^				
Overall	2.15 (0.84)	2.17 (0.99)	.85	73
Primary care	2.02 (0.91)	2.09 (1.06)	.67	42
Physician and APP	2.17 (0.96)	2.10 (0.99)	NA	30
Nurse	2.00 (0.63)	3.00 (1.26)	NA	5
Clinical pharmacist	1.43 (0.49)	1.43 (0.49)	NA	7
Gastroenterology and hepatology	2.32 (0.69)	2.29 (0.89)	.76	31
Physician and APP	2.43 (0.66)	2.57 (0.90)	NA	21
Nurse	2.10 (0.70)	1.70 (0.46)	NA	10
Quality^d^				
Overall	2.59 (0.95)	2.27 (1.10)	.05	73
Primary care	2.73 (0.98)	2.23 (1.12)	.04	42
Physician and APP	2.77 (1.10)	2.27 (1.05)	NA	30
Nurse	2.40 (0.49)	3.20 (1.33)	NA	5
Clinical pharmacist	2.57 (0.49)	1.43 (0.49)	NA	7
Gastroenterology and hepatology	2.48 (0.91)	2.32 (1.06)	.53	31
Physician and APP	2.62 (0.84)	2.71 (1.03)	NA	21
Nurse	2.20 (0.98)	1.50 (0.50)	NA	10
Time^d^				
Overall	2.23 (0.82)	2.29 (1.20)	.55	73
Primary care	2.14 (0.91)	2.19 (1.27)	.60	42
Physician and APP	2.23 (1.02)	2.20 (1.27)	NA	30
Nurse	2.20 (1.33)	3.20 (1.33)	NA	5
Clinical pharmacist	1.71 (0.45)	1.43 (0.49)	NA	7
Gastroenterology and hepatology	2.35 (0.65)	2.42 (1.07)	.81	31
Physician and APP	2.43 (0.58)	2.67 (1.08)	NA	21
Nurse	2.20 (0.75)	1.90 (0.83)	NA	10
Net promoter score^e^				
Overall	NA	10	NA	73
Primary care	NA	17	NA	42
Physician and APP	NA	13	NA	30
Nurse	NA	−60	NA	5
Clinical pharmacist	NA	71	NA	7
Gastroenterology and hepatology	NA	0	NA	31
Physician and APP	NA	−19	NA	21
Nurse	NA	50	NA	10

Abbreviations: APP, advanced practice practitioner; NA, not applicable.

^a^ Physician task load scores range from 0 to 100; lower scores indicate less cognitive task load.^15,16,17^

^b^ Work exhaustion scores range from 0 to 4; lower scores indicate lower levels of work exhaustion.^18,19^

^c^ Two clinicians did not fill out the work exhaustion portion of the presurvey and postsurvey.

^d^ Mean Likert scale scores for utility, quality, and time range from 1 to 5; lower scores indicate a higher level of agreement on a 5-point Likert scale.

^e^ Net promoter score is calculated by categorizing likelihood to recommend responses into promoters (score 9-10), passives (score 7-8), and detractors (score 0-6) and then subtracting the percentage of detractors from the percentage of promoters. The net promoter score ranges from –100 to 100, with higher scores indicating higher levels of satisfaction.^20^

Table 5 showcases themes, representative quotations, and sentiments for the qualitative encoding of free-text survey comments. Comments about draft message voice and/or tone were the most common and included the highest absolute number of negative comments (10 positive, 2 neutral, and 14 negative). The second most common theme was about future use and included the highest absolute and relative number of positive comments (18 positive and 1 negative). The most negative relative comments were about draft message length and/or brevity (1 positive, 2 neutral, and 8 negative). Facilitators for adoption include readiness for future use, utility (13 positive, 2 neutral, and 4 negative), and time-saving (12 positive and 1 negative). Barriers to adoption include draft message voice and/or tone, content relevance (8 positive, 1 neutral, and 9 negative), and accuracy (4 positive and 5 negative).

**Table 5.** Qualitative Encoding of Free-Text Comments From Postsurveys

Theme	Representative quotations	No. of comments			
		Negative	Neutral	Positive	Total
Draft message voice and/or tone	Positive: “I was impressed by the tone that varied based on patient’s concerns and questions, and felt messaging was overall very professional and clear.” Negative: “I think the drafts are great but can further be improved if it did not sound robotic and had a more personable touch.”	14	2	10	26
Future use	Positive: “Please continue to allow us to utilize this tool and spread to other SHC clinics!” Negative: “I still think it’s a good idea but not ready for real life situations.”	1	0	18	19
Draft message tool utility	Positive: “Overall this is a very helpful tool.” Negative: “Also, it struggled with having draft replies of more nuanced concerns.”	4	2	13	19
Draft message content relevance	Positive: “I especially appreciated the one example where a patient mentioned having frequent UTIs on a certain medication, and the response had pulled in the last 3 lab results from urinalysis!” Negative: “The Reponses often did not accurately reflect the questions. Sometimes way off. Often vague.”	9	1	8	18
Impact on workflow	Positive: “It helped with the ‘translation’ cognitive work that I hadn’t ever realized I was doing before process of translating my medical understanding into patient-facing language.” Negative: “I have to read the actual draft before starting to work on the actual request, as I don’t know if the response is even appropriate.”	9	0	7	16
Impact on time	Positive: “It helped save me a lot of time starting from scratch.” Negative: “Right now, it is just piling on top of the work that we are already doing, and it is faster for me to type a prose response that I have generated myself.”	1	0	12	13
Draft message length and/or brevity	Positive: “However, the responses are very thorough. I had a patient that needed a refill and the draft wrote out almost a whole letter when I typically would maybe just write a short sentence saying ‘Yes, I will send!’” Negative: “Overall the responses seemed unnecessarily wordy in noncontributory ways.”	8	2	1	11
Draft message content accuracy	Positive: “I found the AI-generated draft replies pretty accurate and helpful.” Negative: “Sometimes, the AI response was not completely accurate, but it was not difficult to make minor tweaks to the draft.”	5	0	4	9
Impact on patient engagement	Positive: “This may have a positive impact on patient satisfaction with longer messages.” Negative: “Patients can tell these responses were AI generated, they are formatted like the AI responses we get on airline websites.”	2	2	3	7
Draft message content completeness	Positive: “Good things are AI can capture all the elements in the message patient sent and address each element.” Negative: “The AI responses were a great initial draft, though often required some additional information or editing.”	4	0	1	5
Total	NA	57	9	77	143

Abbreviations: AI, artificial intelligence; NA, not applicable; SHC, Stanford Health Care; UTI, urinary tract infection.

## Discussion

Although generative AI may ultimately transform the practice of medicine and help address challenges in care delivery, it is important to ground organizational strategy in clinical practice data about outcomes and value. In one of the earliest implementations of generative AI in clinical practice, this quality improvement study evaluated the adoption, usability, and utility of AI-generated draft replies to patient messages. The mean cumulative draft utilization after only 5 weeks was 20%. This is remarkable given that (1) these versions of GPT were not trained on medical literature or fine-tuned for this task specifically, (2) limited context was available from the patient’s EHR for draft generation, and (3) minimal user education was necessary for adoption.

Improvements in task load and emotional exhaustion scores suggest that generated draft replies have the potential to impact cognitive burden and burnout. Similarly, users expressed high expectations about utility, quality, and time that were either met or exceeded at the end of the pilot. Given the evidence that burnout is associated with turnover, reductions in clinical activity, and quality, even a modest improvement may have a substantial impact.^21,22,23,24^

Despite improvements in burden and burnout, no changes in overall reply time, read time, or write time were found when comparing prepilot and pilot periods. It may be that switching from writing to editing may be less cognitively taxing despite taking the same amount of time. That said, survey respondents showed optimism about time saved, suggesting that perceptions of time may be different from time captured via EHR metadata, both of which may be different than real time. Finally, although future iterations may focus on measurable time-savings, considering other relevant outcomes including volume of follow-up messages and patient experience will provide a more complete picture.

Subgroup analysis showed that gastroenterology and hepatology nurses had higher draft utilization, a trend toward time saved, and positive net promoter scores. This finding suggests that the value of future generative AI tools may vary according to specific practice patterns and workflows. Understanding this fit is necessary to optimize use, especially in cases where tools are expensive or require substantial training. In addition to between-group heterogeneity, there was also significant within-group heterogeneity for adoption and time spent. These findings were supported by the qualitative analysis of free-text feedback where, for example, some individuals preferred longer, more empathetic responses but others preferred shorter, more formal responses.

Given the rapid evolution of generative AI, these shortcomings represent opportunities for further research and development. Personalization can be addressed at the vendor level (eg, embedded tools to control brevity and tone) or at the system level (fine-tuning on data specific to an institution or individuals). Additional enhancements may also lead to improved performance, including increased access to patient information to inform draft generation, optimized categorization leading to better response specificity, and the development of large language models trained and/or fine-tuned on medical literature and EHR data.

Finally, it is worth considering the need for reference standards. Although there has been early work done to identify benchmarks for training sets for EHR data,^25^ there is no reference standard for patient message replies either from a clinician or patient perspective. As generative AI technologies evolve, identifying reference standards and developing training sets to fine-tune models and evaluate model performance for specific use cases will be essential.

### Limitations

This was a single-group prospective study at a single institution, which limits the generalizability of results. Although there were 162 participants in the primary analyses and 73 survey respondents in the supplemental analyses, they were limited to 2 departments and 4 types of clinicians. Given the observed between-group and within-group heterogeneity, the study may have been insufficiently powered to identify subgroup differences. Regarding cognitive burden and burnout outcomes, novelty bias and the Hawthorne effect may have skewed toward positive results. Regarding time, there are known limitations to the use of EHR metadata to approximate time,^26^ which underscores the need to develop standardized time calculations and definitions. Future controlled trials at multiple sites with a focus on understanding what might be mediating the changes identified in this study are warranted.

## Conclusions

In this quality improvement study of generative AI in health care, using GPT-4 to generate draft responses to patient messages, we found adoption, usability, and notable improvements in mental task load and work exhaustion. There were no adverse safety signals, and qualitative feedback suggested high expectations for future use. These findings are especially remarkable given minimal user education and the use of a large language model without domain-specific training. That said, we did not find time-savings, and feedback highlighted the need for improvements in tone, brevity and personalization. Ongoing code-to-bedside testing is needed to inform future development and strategic organizational decision-making. In the case of generated draft messages, there is a cost each time GPT-4 is used to generate a draft response; multiplying that cost by millions of patient messages could represent a substantial expense to the US health care delivery system that must be justified with clinical practice data. Although the transformative potential of generative AI is evident, understanding when these tools have reached a maturity level to warrant costly investments and widespread use is paramount.

## Notes

Supplement 1. **eFigure 1.** Large Language Model Integration **eFigure 2.** Mock User Interface **eTable 1.** Application of RE-AIM/PRISM for Evaluation of Implementation **eTable 2.** Other Action Inclusions and Exclusions **eFigure 3.** Time Metric Definitions **eFigure 4.** Pre- and Postsurvey Questionnaire **eFigure 5.** Clinician Recruitment Flowchart **eFigure 6.** Histogram of Draft Utilization per Clinician With Pareto **eFigure 7.** Draft Utilization vs Reply Action Count per Clinician **eTable 3.** Draft Availability for All Unique Messages Viewed, Acted on or Replied to **eFigure 8.** Average Time Spent per Clinician on Actions in Prepilot vs Pilot Periods **eFigure 9.** Average Time Spent on Actions in Prepilot vs Pilot Periods Using a Linear Mixed Effects Model **eTable 4​.** Impact of Draft Messages on Change in Log-Transformed Time Spent, Analyzed by Linear Mixed Effects Models

Supplement 2.
Data Sharing Statement
