# Supplementary material for: Artificial Intelligence–Generated Draft Replies to Patient Inbox Messages
Source: JAMA Netw Open. 2024 Mar 20;7(3):e243201. doi: 10.1001/jamanetworkopen.2024.3201 (PMC10955355; doi:10.1001/jamanetworkopen.2024.3201)
Supplement: Supplement 1. — eFigure 1. Large Language Model Integration eFigure 2. Mock User Interface eTable 1. Application of RE-AIM/PRISM for Evaluation of Implementation eTable 2. Other Action Inclusions and Exclusions eFigure 3. Time Metric Definitions eFigure 4. Pre- and Postsurvey Questionnaire eFigure 5. Clinician Recruitment Flowchart eFigure 6. Histogram of Draft Utilization per Clinician With Pareto eFigure 7. Draft Utilization vs Reply Action Count per Clinician eTable 3. Draft Availability for All Unique Messages Viewed, Acted on or Replied to eFigure 8. Average Time Spent per Clinician on Actions in Prepilot vs Pilot Periods eFigure 9. Average Time Spent on Actions in Prepilot vs Pilot Periods Using a Linear Mixed Effects Model eTable 4​. Impact of Draft Messages on Change in Log-Transformed Time Spent, Analyzed by Linear Mixed Effects Models [file jamanetwopen-e243201-s001.pdf]

Supplemental Online Content

Garcia P, Ma SP, Shah S, et al. Artificial intelligence–generated draft replies to patient inbox messages. *JAMA Netw Open*. 2024;7(3):e243201. doi:10.1001/jamanetworkopen.2024.3201

eFigure 1. Large Language Model Integration

eFigure 2. Mock User Interface

eTable 1. Application of RE-AIM/PRISM for Evaluation of Implementation

eTable 2. Other Action Inclusions and Exclusions

eFigure 3. Time Metric Definitions

eFigure 4. Pre- and Postsurvey Questionnaire

eFigure 5. Clinician Recruitment Flowchart

eFigure 6. Histogram of Draft Utilization per Clinician With Pareto

eFigure 7. Draft Utilization vs Reply Action Count per Clinician

eTable 3. Draft Availability for All Unique Messages Viewed, Acted on or Replied to

eFigure 8. Average Time Spent per Clinician on Actions in Prepilot vs Pilot Periods

eFigure 9. Average Time Spent on Actions in Prepilot vs Pilot Periods Using a Linear Mixed Effects Model

eTable 4. Impact of Draft Messages on Change in Log-Transformed Time Spent, Analyzed by Linear Mixed Effects Models

This supplemental material has been provided by the authors to give readers additional information about their work.

© 2024 Garcia P et al. *JAMA Network Open.*

eFigure 1. Large Language Model Integration

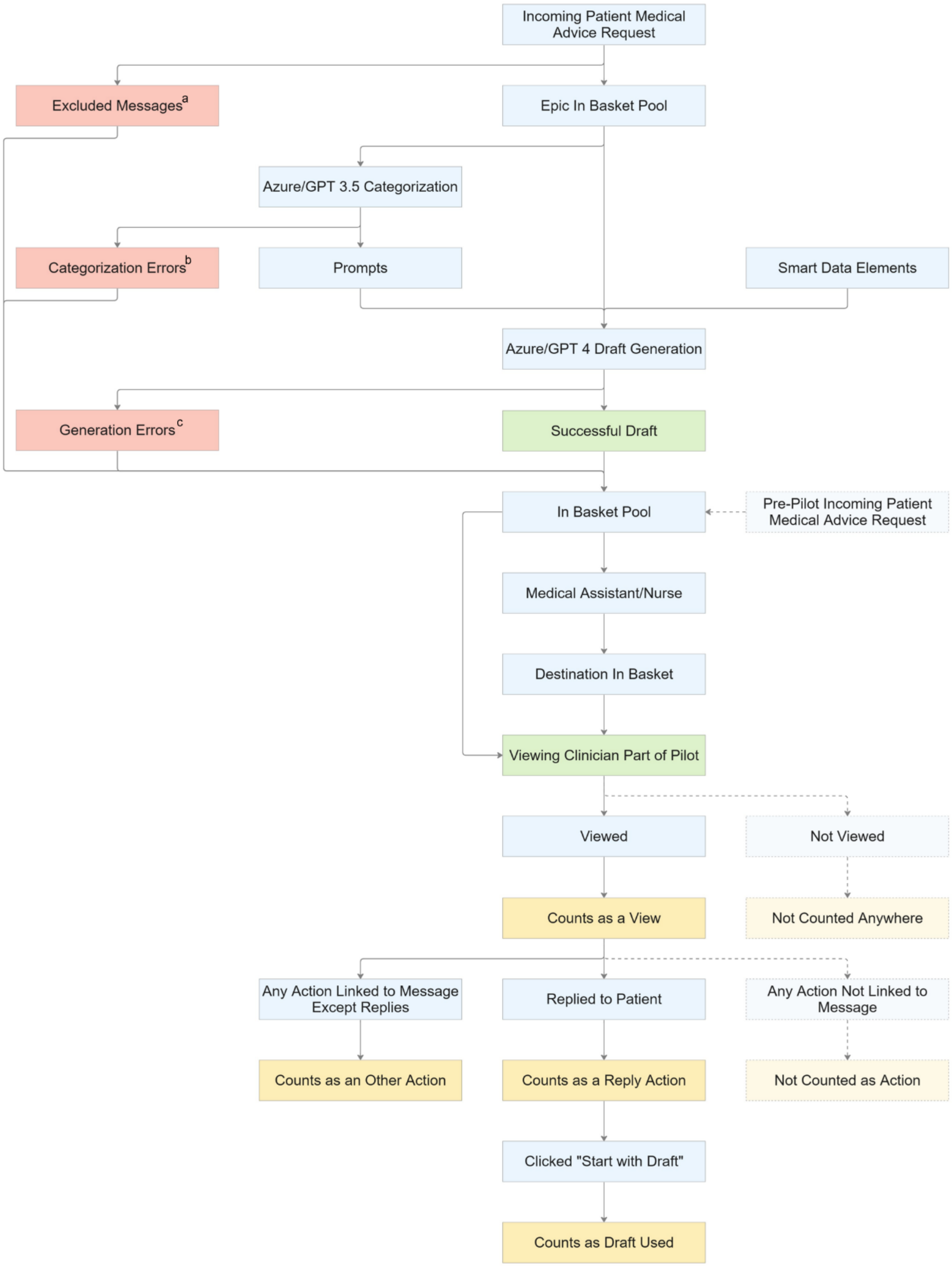

a, Excluded messages include: 1) recipient not part of the pilot, 2) non-English language, 3) contains attachment, 4) sender was a proxy
b, Categorization errors include: 1) insufficient compute (*HTTP timeout was hit for OpenAI, Request to LLM timed out*)
c, Generation errors include: 1) prompt or message exceeds token limit (*Max model length was reached, Response cutoff due to max length limits*), 2) insufficient compute (*HTTP timeout was hit for OpenAI, Request to LLM timed out*), 3) prompt or message filtered for inappropriate content (*Prompt was filtered, Response to prompt was filtered*), 4) communication failure between Azure and language model (*API error*)

© 2024 Garcia P et al. *JAMA Network Open.*

eFigure 2. Mock User Interface
© 2024 Epic Systems Corporation. Used with permission.

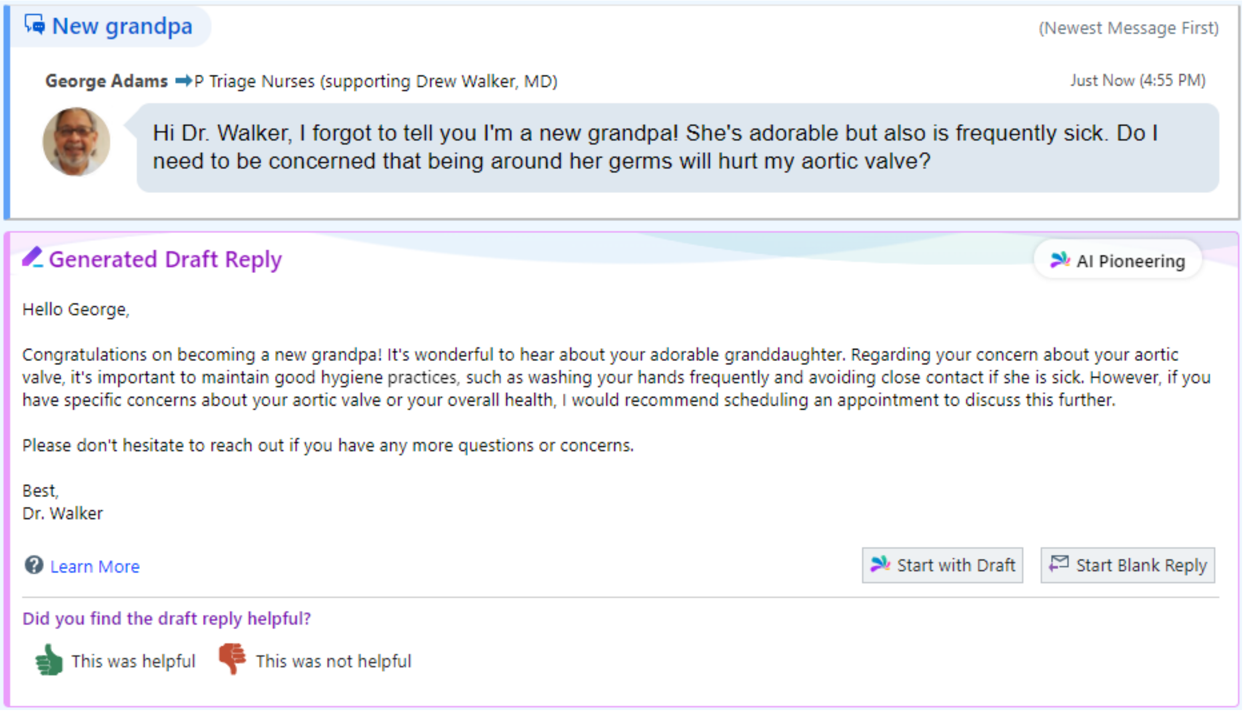

© 2024 Garcia P et al. *JAMA Network Open.*

eTable 1. Application of RE-AIM/PRISM for Evaluation of Implementation

Category	Data Source	Study Measure
Reach	EHR	Volume of providers in pilot
		Volume of messages sent
		Draft Utilization
Effectiveness	EHR	Time
	Surveys	Burden
		Burnout and professional fulfillment
Adoption	Surveys	Barriers to/facilitators of adoption
Implementation	Surveys	Barriers to/facilitators of implementation
Clinician Characteristics	Surveys	Demographic characteristics of participating clinicians
External Environment	Surveys	National healthcare context (burnout)
Clinician Perspectives on the Intervention	Surveys	NPS
		AI tool usability
		AI tool accuracy
Sustainability and Maintenance Infrastructure	Surveys	Barriers to/facilitators of maintenance and sustainability

© 2024 Garcia P et al. *JAMA Network Open.*

eTable 2. Other Action Inclusions and Exclusions

Criteria	Code	Action
Inclusion	R HIC 102	REPLY TO SENDER
Inclusion	R HIC 106	FORWARD MESSAGE
Inclusion	R HIC 114	READ MESSAGE
Inclusion	R HIC 115	PEND MESSAGE
Inclusion	R HIC 118	REPLY TO ALL
Inclusion	R HIC 155	TAKE RESPONSIBILITY
Inclusion	R HIC 156	PUT RESPONSIBILITY BACK
Inclusion	R HIC 161	RECALL MESSAGE
Inclusion	R HIC 162	MOVE TO MY MESSAGES
Inclusion	R HIC 163	IB FORWARD MESSAGES
Inclusion	R HIC 1018	FOLLOW UP
Inclusion	R HIC 1019	GENERAL PROPERTIES – NO FOLLOWUP
Inclusion	R HIC 2004	PATIENT REVIEW
Inclusion	R HIC 2005	CREATE TELEPHONE CALL
Inclusion	R HIC 2155	TAKE PUT BACK RESPONSIBILITY SUBMENU
Inclusion	R HIC 3210	MYCHART – HYPERSPACE ENCOUNTER CREATION
Inclusion	R HIC 3216	PLACE ORDER AFTER OPENING MYCHART ENCOUNTER
Inclusion	R HIC 3240	MYCHART – NO ACTION REQUIRED MESSAGE HANDLED
Inclusion	R HIC 3241	MYCHART – CALLED PATIENT MESSAGE HANDLED
Inclusion	R HIC 8999	UNDEFER TO WORKSTATION
Inclusion	R HIC 9000	DEFER TO WORKSTATION
Inclusion	R HIC 9511	IB DISMISS LPG INTRO
Inclusion	R HIC 9550	IB SUGGESTED RESPONSE
Inclusion	R HIC 9551	IB SUGGESTED RESPONSE IS USEFUL
Inclusion	R HIC 9552	IB SUGGESTED RESPONSE DEFICIENCIES
Inclusion	R HIC 210101301	SHC AMB POSTPONE MESSAGE – TOMORROW
Inclusion	R HIC 210101302	SHC AMB POSTPONE MESSAGE – 3 DAYS
Inclusion	R HIC 210101303	SHC AMB POSTPONE MESSAGE – 7 DAYS
Inclusion	R HIC 210101304	SHC AMB POSTPONE MESSAGE – CHOOSE DATE
Inclusion	R HIC 210101305	SHC AMB POSTPONE MESSAGE – CHOOSE DATE DEFAULT
Inclusion	R HIC 210101306	SHC AMB POSTPONE MESSAGE – 2 DAYS
Exclusion	R HIC 104	VIEW REPORT
Exclusion	R HIC 116	MARK AS NEW
Exclusion	R HIC 125	RETRACT MESSAGE
Exclusion	R HIC 128	EDIT OUT BASKET
Exclusion	R HIC 129	FORWARD OUT BASKET
Exclusion	R HIC 131	CLEAR ONLY-SENT MESSAGES
Exclusion	R HIC 132	UNDO CLEAR-SENT MESSAGES

© 2024 Garcia P et al. *JAMA Network Open.*

eFigure 3. Time Metric Definitions

Metric	Start Message View	Other Action Started	
Other Action Time	Start Interval	End Interval	

Metric	Start Message View	Clicked on "Reply to Patient", "Start Blank Reply", or "Start with Draft”	Message Sent
Reply Action Time	Start Interval		End Interval
Read Time	Start Interval	End Interval	
Write Time		Start Interval	End Interval

© 2024 Garcia P et al. *JAMA Network Open.*

eFigure 4. Pre- and Postsurvey Questionnaire

Pre-Measurement Survey	Post-Measurement Survey
**Physician Task Load Derivative Index**<br><br>Please reflect on a day that you responded to patient messages in the last 1-2 weeks that is representative of a typical current day responding to patient messages, and move the sliders to indicate your response (Very Low, Low, Medium, High, Very High; 0-100 continuous):<br>• How mentally demanding was the task of responding to patient messages?<br>• How hurried or rushed was the pace of responding to patient messages?<br>• How hard did you have to work to achieve your level of performance responding to patient messages?<br>• How physically demanding was the task of responding to patient messages?	**Physician Task Load Derivative Index**<br><br>Please reflect on a day that you responded to patient messages in the last 1-2 weeks that is representative of a typical current day responding to patient messages using the AI-generated draft replies tool, and move the sliders to indicate your response (Very Low, Low, Medium, High, Very High; 0-100 continuous):<br>• How mentally demanding was the task of responding to patient messages?<br>• How hurried or rushed was the pace of responding to patient messages?<br>• How hard did you have to work to achieve your level of performance responding to patient messages?<br>• How physically demanding was the task of responding to patient messages?
**Burnout and Emotional Exhaustion Score**<br><br>To what degree have you experienced the following? (0-4; Not at all, Very Little, Moderately, A Lot, Extremely) During the past two weeks, I have felt...<br>• A sense of dread when I think about work I have to do.<br>• Physically exhausted at work.<br>• Lacking in enthusiasm at work.<br>• Emotionally exhausted at work.	**Burnout and Emotional Exhaustion Score**<br><br>To what degree have you experienced the following? (0-4; Not at all, Very Little, Moderately, A Lot, Extremely) During the past two weeks, I have felt...<br>• A sense of dread when I think about work I have to do.<br>• Physically exhausted at work.<br>• Lacking in enthusiasm at work.<br>• Emotionally exhausted at work.
**Anticipated Utility**<br><br>In your opinion, what is the likelihood that AI-generated draft replies embedded in the EHR in-basket would...<br>• be useful to respond to patient messages.<br>• decrease the amount of time it takes to respond to patient messages.<br>• improve the quality of my responses to patient messages.<br>• would draft responses to patient messages in the appropriate voice/tone.	**Utility**<br><br>Based on your experience using AI-generated draft replies embedded in the EHR in-basket, please indicate how much you agree or disagree with the following statements:<br>• The AI-generated draft replies were useful when responding to patient messages.<br>• The AI-generated draft replies decreased the amount of time it takes to respond to patient messages.<br>• The AI-generated draft replies improved the quality of my responses to patient messages.<br>• The AI-generated draft replies were written in the appropriate voice/tone.
	**Net Promotor**<br><br>How likely are you to recommend using the AI-generated draft replies tool to respond to patient messages to a colleague? (0-10)

© 2024 Garcia P et al. *JAMA Network Open.*

	**Free-Text Response**<br><br>Please provide any additional comments or feedback regarding the utility and workflow impact of the AI-generated draft replies tool (free-text).

© 2024 Garcia P et al. *JAMA Network Open.*

eFigure 5. Clinician Recruitment Flowchart

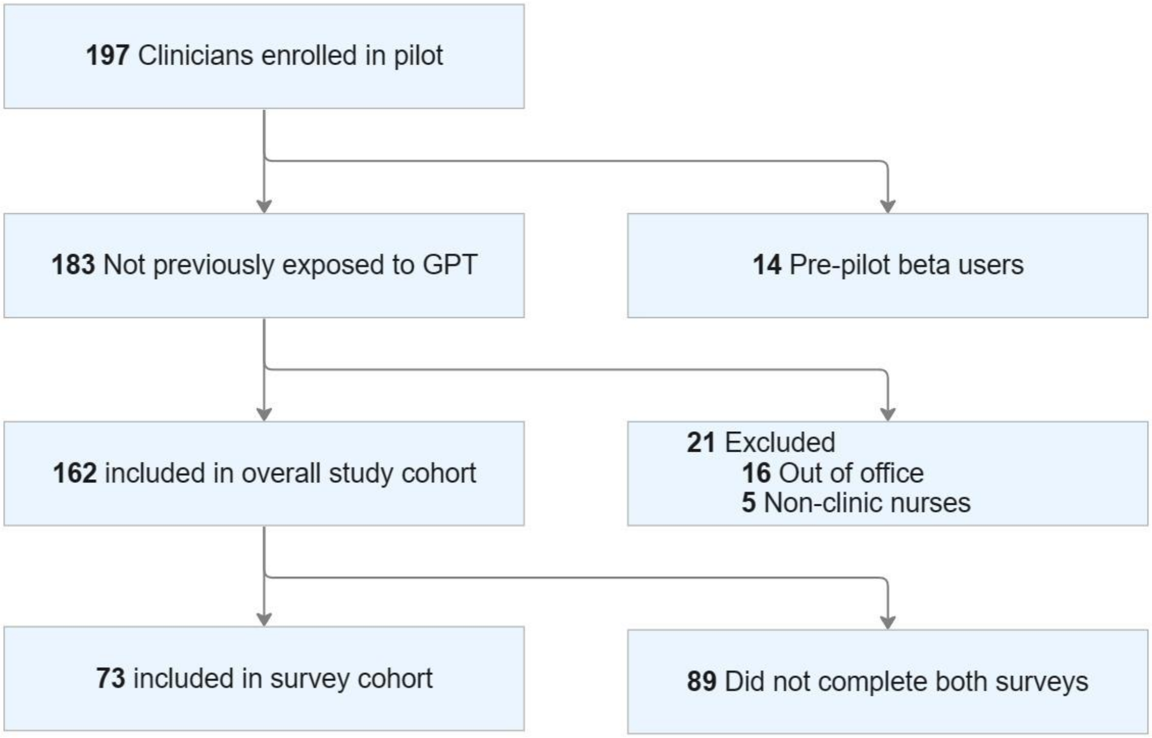

© 2024 Garcia P et al. *JAMA Network Open.*

eFigure 6. Histogram of Draft Utilization per Clinician With Pareto

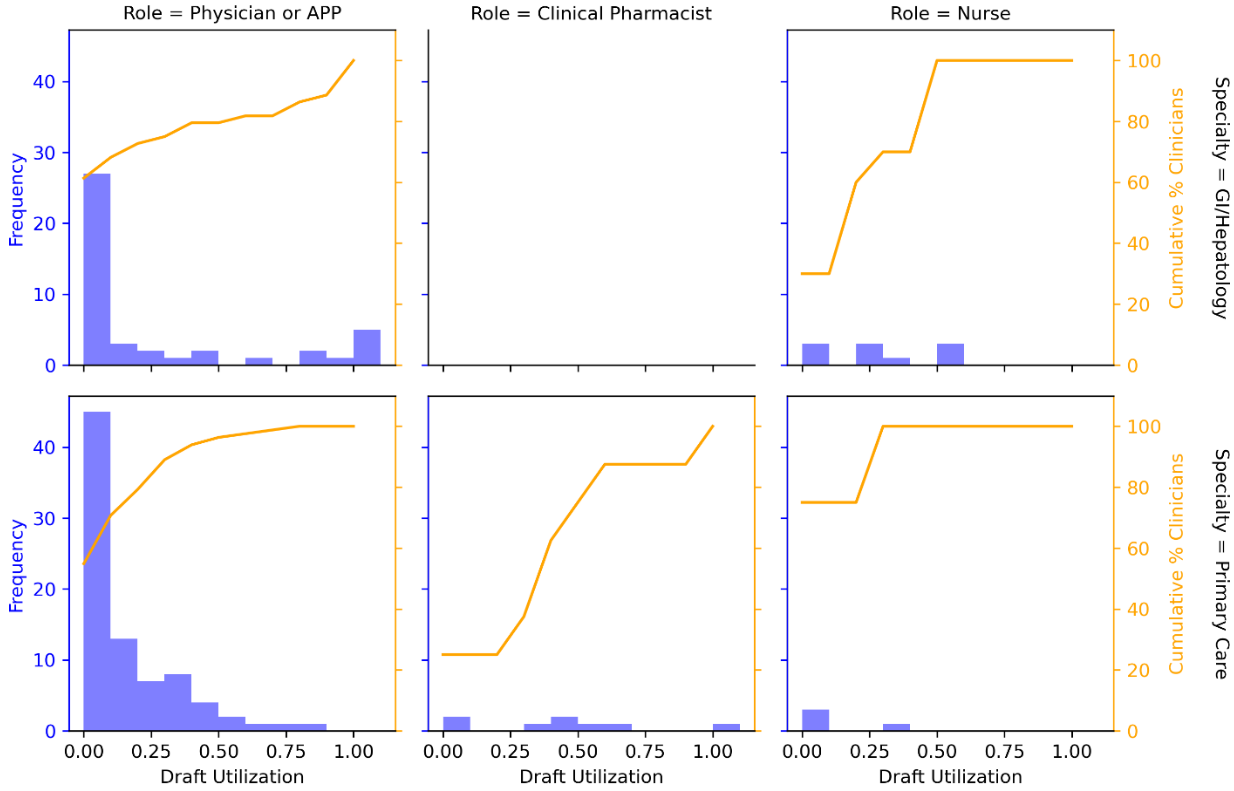

© 2024 Garcia P et al. *JAMA Network Open.*

eFigure 7. Draft Utilization vs Reply Action Count per Clinician

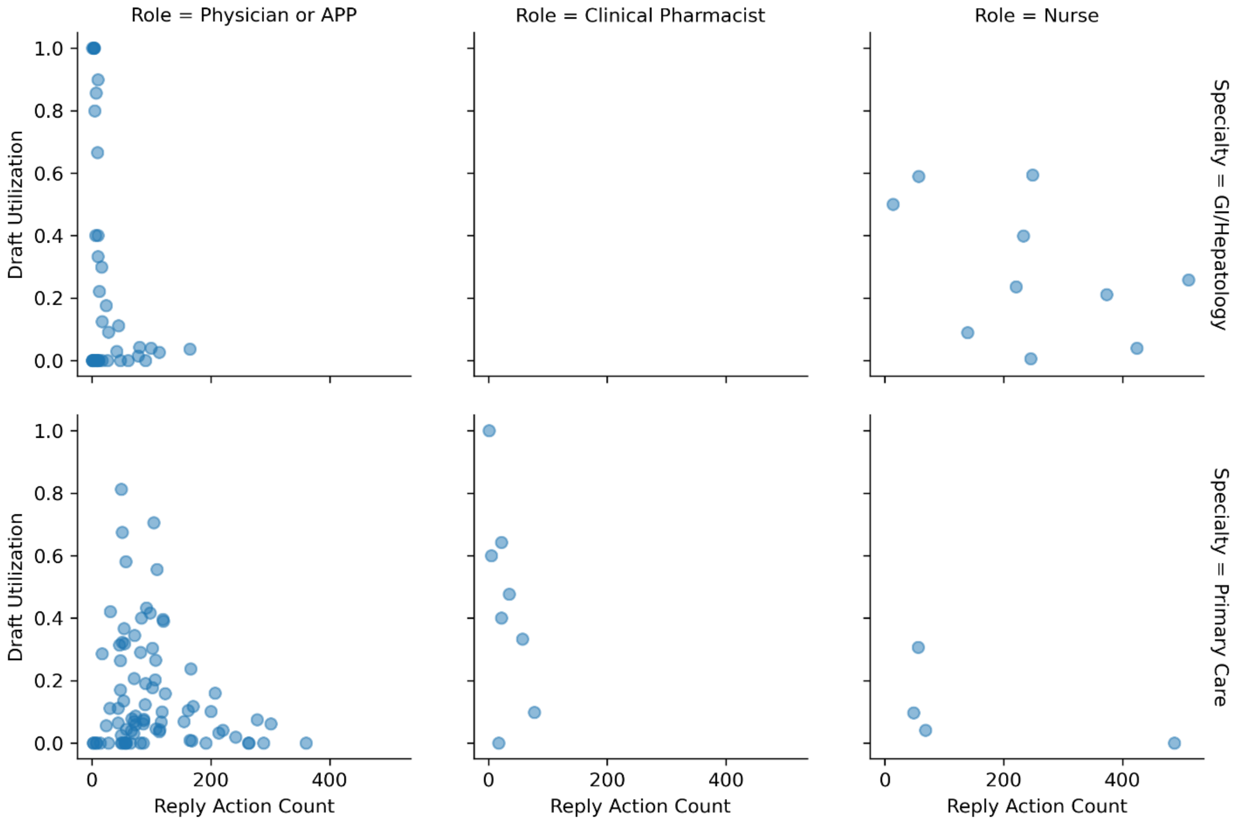

© 2024 Garcia P et al. *JAMA Network Open.*

eTable 3. Draft Availability for All Unique Messages Viewed, Acted on or Replied to

Metric	Category	Counts	Percentages
**Messages with Views**	**Total**	**32829**	**100.00%**
	Drafts Available	23164	70.56%
**Messages with Other Actions**	**Total**	**15694**	**100.00%**
	Drafts Available	10679	68.05%
**Messages with Patient Replies**	**Total**	**12844**	**100.00%**
	Drafts Available	9621	74.91%
	Excluded Messages	2596	20.21%
	Categorization Errors	389	3.03%
	HTTP timeout was hit for OpenAI	*282*	*2.20%*
	Request to LLM timed out	*107*	*0.83%*
	Generation Error	238	1.85%
	Max model length was reached	*128*	*1.00%*
	API error	*42*	*0.33%*
	HTTP timeout was hit for OpenAI	*25*	*0.19%*
	Response cutoff due to max length limits	*21*	*0.16%*
	Prompt was filtered	*10*	*0.08%*
	Request to LLM timed out	*8*	*0.06%*
	Response to prompt was filtered	*4*	*0.03%*

© 2024 Garcia P et al. *JAMA Network Open.*

eFigure 8. Average Time Spent per Clinician on Actions in Prepilot vs Pilot Periods

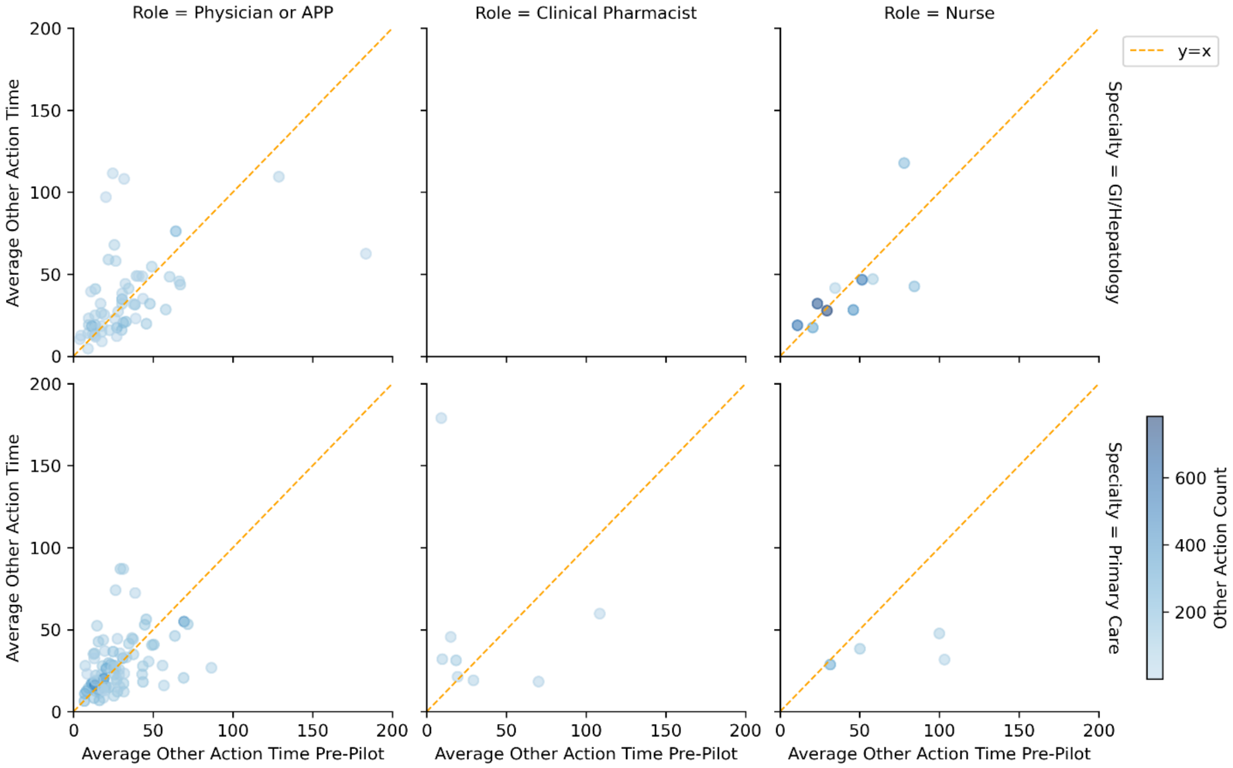

© 2024 Garcia P et al. *JAMA Network Open.*

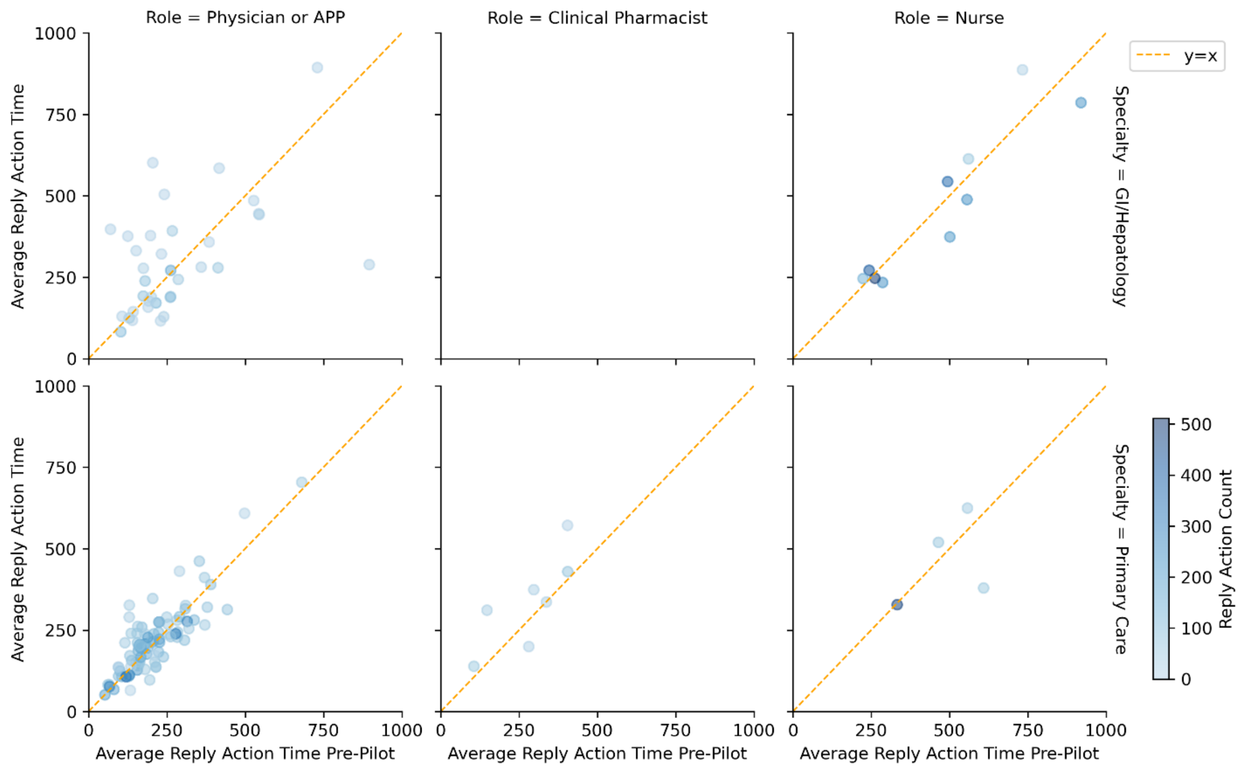

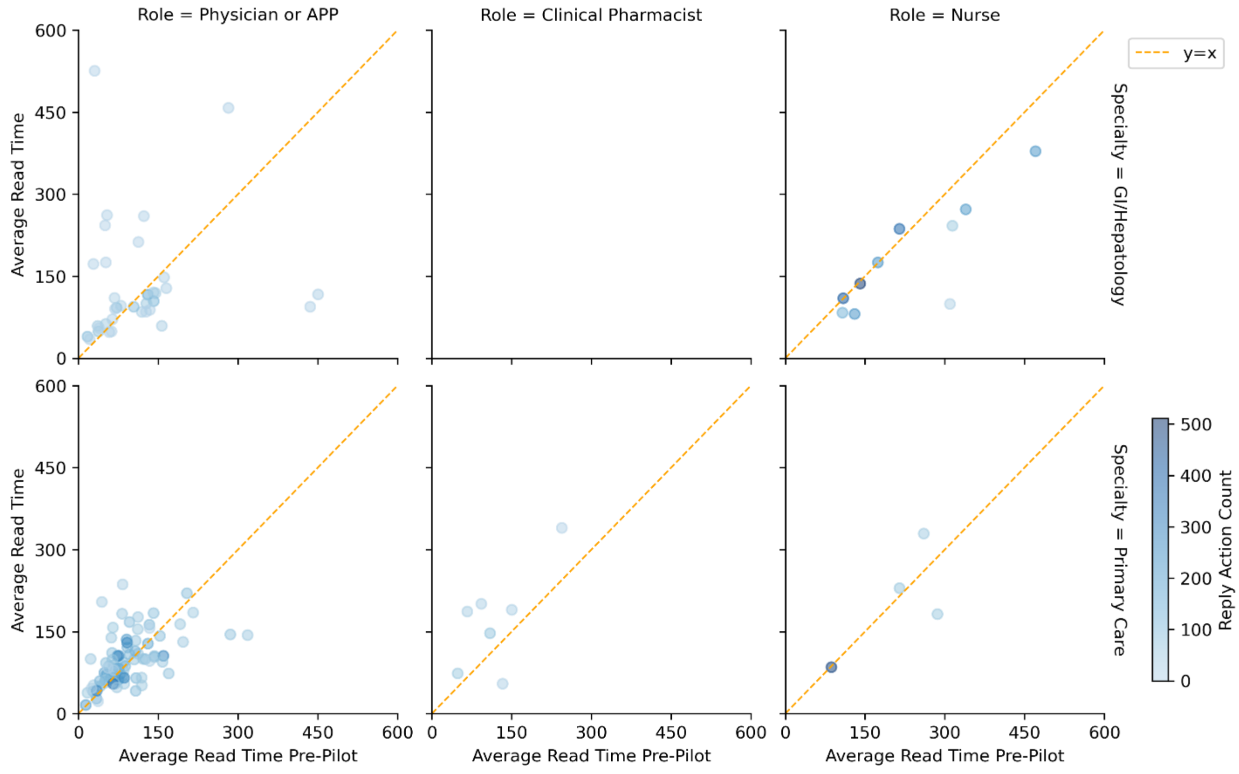

© 2024 Garcia P et al. *JAMA Network Open.*

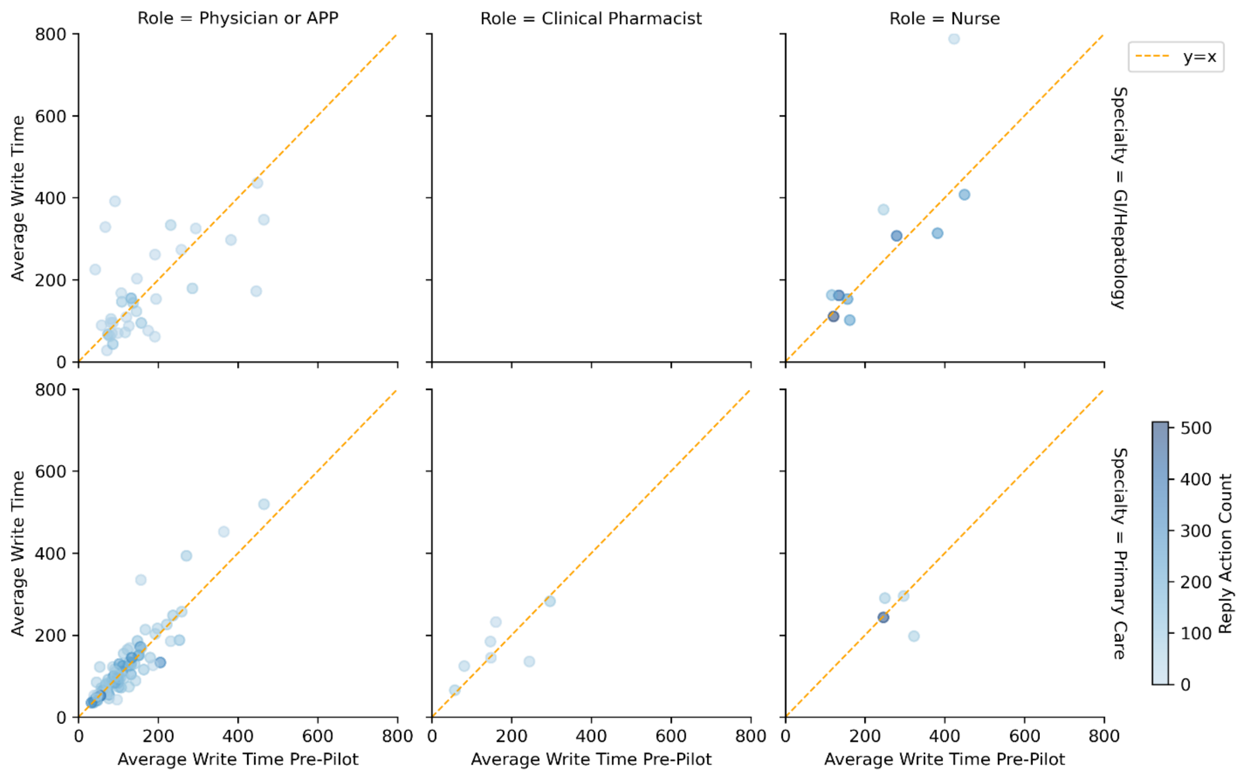

© 2024 Garcia P et al. *JAMA Network Open.*

eFigure 9. Average Time Spent on Actions in Prepilot vs Pilot Periods Using a Linear Mixed Effects Model

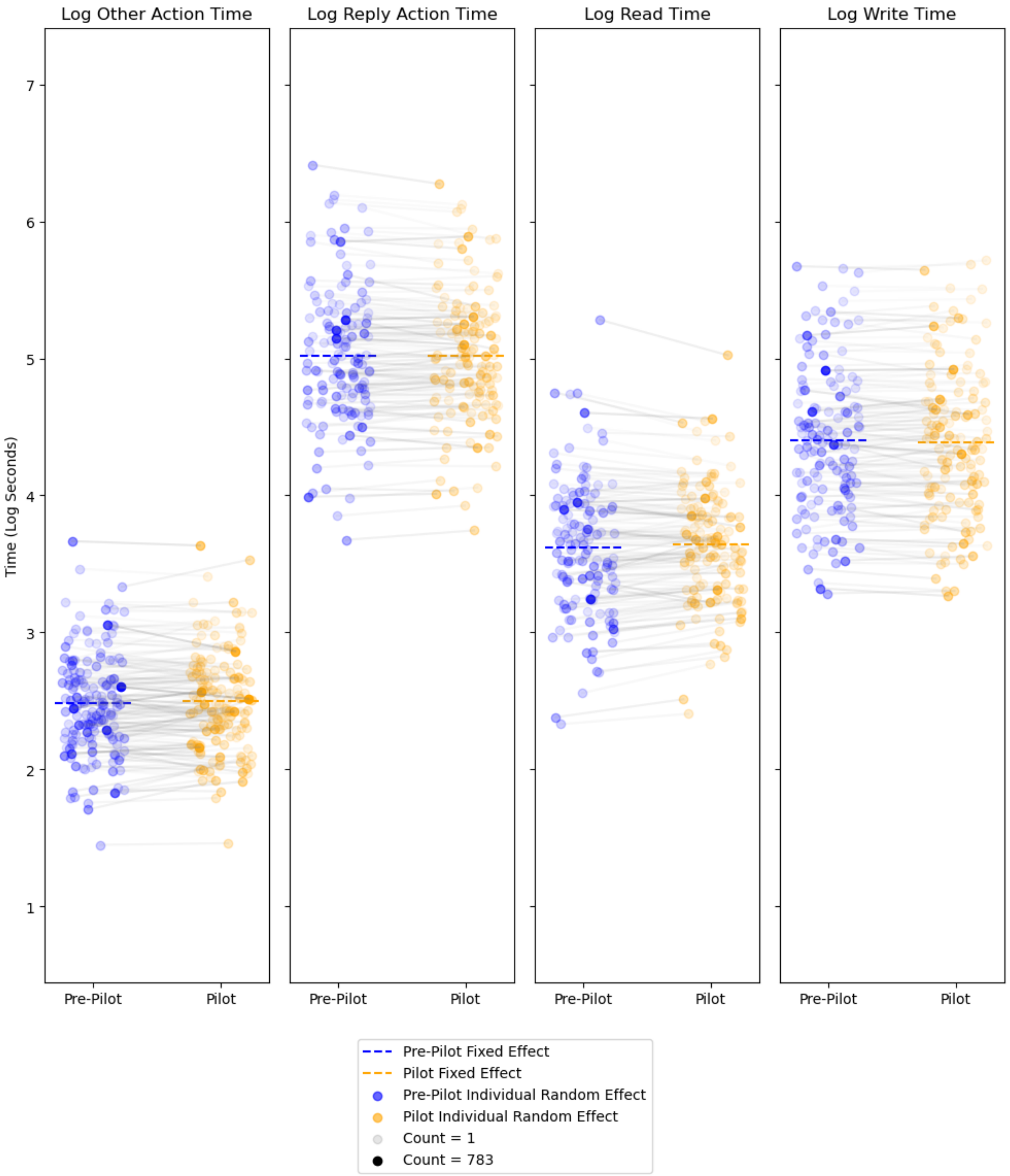

© 2024 Garcia P et al. *JAMA Network Open.*

eTable 4. Impact of Draft Messages on Change in Log-Transformed Time Spent, Analyzed by Linear Mixed Effects Models

Metric	No. of observations	No. of groups (clinicians)	Mean group size (messages per clinician)	Intervention coefficient (change in time, log(s))	Coefficient Standard Error	p-value
Other Action Time	32153	161	199.7	0.014	0.035	0.475
Reply Action Time	24847	138	180.1	-0.003	0.017	0.859
Read Time	-	-	-	0.020	0.025	0.427
Write Time	-	-	-	-0.008	0.018	0.651

© 2024 Garcia P et al. *JAMA Network Open.*
