# Supplementary material for: Artificial Intelligence–Generated Draft Replies to Patient Inbox Messages
Source: JAMA Netw Open. 2024 Mar 20;7(3):e243201. doi: 10.1001/jamanetworkopen.2024.3201 (PMC10955355; doi:10.1001/jamanetworkopen.2024.3201)
Supplement: Supplement 2. — Data Sharing Statement [file jamanetwopen-e243201-s002.pdf]

Data Sharing Statement

Garcia. Artificial Intelligence–Generated Draft Replies to Patient Inbox Messages. *JAMA Netw Open*. Published March 20, 2024. doi:10.1001/jamanetworkopen.2024.3201

Data
Data available: No
